# Treatment of Diphtheria

**Published:** 1868-06-15

**Authors:** James C. Bascomb

**Affiliations:** McLean, Ill.


### TREATMENT OF DIPHTHERIA.

*To the Editor of the Chicago Medical Journal :*

Dear Sir :—In the May number of the Journal is an article from the pen of Professor Miller, which I fully endorse, for the reason that I have been practicing on the principles therein contained, and using the same treatment, for the past five years, in diphtheria, with an almost universal success, having treated seven hundred well marked cases of diphtheria, of all ages and conditions, with but a loss of ten cases. One year ago last winter I wrote a short article to the Journal, giving this exact view of the pathology and treatment of this formidable disease, but I suppose it was so directly opposed to the then received views of the profession, and the author being unknown and obscure, the communication never appeared. I was led to adopt this course of treatment by studying the pathology of the disease, assisted by old Dr. Polin, of Springfield, Ky., in a wide spread epidemic of this then little understood disease. My treatment has been adopted by my brethren of this village, and by a student of mine who graduated at Rush College last winter, Dr. R. N. Barger, with the same invariable success; and I write this with the hope of adding some weight to the testimony of Dr. Miller, and correcting the egregious errors of the profession generally, in regard to this disease. I only differ from Dr. Miller in giving more *Chlorate of Potash*, dissolving it in hot water, and give all the stomach will bear, leaving out the *Simple syrup*, believing it to have a better local effect, and applying on the outside a warm, moist bag of salt. I have had several cases of paralysis as a sequela of this disease, which in every instance has been arrested by *Strychnine*.

Respectfully, JAMES C. BASCOM.

MCLEAN, ILL., *June 5th*, 1868.

[NOTE BY THE EDITOR.—We have no recollection of receiving the article alluded to by our friend, Dr. Bascom. These is, of course, no particular novelty about this mode of treatment. Numberless practitioners have approximated it. Prof. Miller's article, and the above, are valuable as cumulative evidences of a form of successful treatment, differing, *toto cœlo*, from that suggested by DR. CONDIE, in his generally valuable work on Diseases of Children, of which notice was taken some time since.]

